# Immigrant Status and Ethnic Inequities in Dental Caries in Children: Bilbao, Spain

**DOI:** 10.3390/ijerph19084487

**Published:** 2022-04-08

**Authors:** Elena Rodriguez-Alvarez, Luisa N. Borrell, Elena Marañon, Nerea Lanborena

**Affiliations:** 1 Department of Nursing I, University of the Basque Country (UPV/EHU), 48940 Leioa, Bizkaia, Spain; elena.maranon@ehu.eus (E.M.); nerea.lamborena@ehu.eus (N.L.); 2 OPIK-Research Group for Social Determinants of Health and Demographic Change, University of the Basque Country (UPV/EHU), 48940 Leioa, Bizkaia, Spain; luisa.borrell@sph.cuny.edu; 3 Department of Epidemiology & Biostatistics, Graduate School of Public Health & Health Policy, City University of New York, New York, NY 10027, USA; 4 Department of Surgery, Medical and Social Science, University of Alcala, 28871 Madrid, Spain; 5 Health Promotion, Health and Consumption Area of Bilbao City Council, 48007 Bilbao, Spain

**Keywords:** dental caries, children, immigration, ethnicity, inequities, survey

## Abstract

This study examined the migratory status/ethnic inequities in dental caries in school children aged 4–9 years (*n* = 1388) and the impact of the Children’s Oral Health Program in the Municipality of Bilbao in the Basque Country Region, Spain. Using the 2017 Children’s Oral Health Survey, log binomial regression was used to quantify the association of parental immigration status/ethnicity with tooth decay for (1) the primary and the permanent dentitions, separately, in children 4–9 years old; and (2) for the permanent dentition in children aged 7–9 years. Compared with Spanish children, Spanish Roma and immigrant children had a higher probability of tooth decay in primary and permanent teeth after adjustment. Similarly, Spanish Roma and immigrant children had a higher probability of caries experience in primary and permanent teeth. In children aged 7–9 years, Spanish Roma children had a greater probability of tooth decay and caries experience (*DMFT* index ≥ 1; PR: 6.20; 95% CI: 3.18, 12.12; and PR: 4.52; 95% CI: 2.46, 8.32; respectively) compared with Spanish Children. These associations were not observed in immigrant children. This study shows that parental immigration status and/or ethnicity affect caries outcomes in immigrant and Roma children in both primary and permanent dentition.

## 1. Introduction

Dental caries is one of the most common chronic diseases in the world [1], and the foremost in childhood, affecting 50% of children between the ages of 2 and 5 years [2]. Furthermore, it is estimated that 10% of dental caries are untreated, which translates into a significant burden of disease [3]. In fact, several studies describe the relationship of dental caries in the primary and permanent dentitions with malnutrition [4], growth retardation [5] as well as in learning due to the loss of school days [6]. These conditions could have lasting effects given the important relationship of health in the early years of life with implications for a long and healthy life in adulthood [7]. Although in the last three decades, childhood caries, especially in permanent teeth, has decreased worldwide [8], parental migratory status and/or ethnic inequities in dental caries is pervasive [9,10,11] and has even increased [12]. Thus, children of immigrant origin and/or ethnic minorities make up a particularly vulnerable population group in various health indicators, including oral health [13]. To that end, parental migratory and/or ethnic status are considered an axis of inequities in health that exerts its influence beyond one generation [14].

In Europe, inequities in childhood dental caries based on parental migratory status are evident, although they vary when considering the country of origin and of destination [15,16]. The latter may be a consequence of the differences in access to dental services provided by the different health systems in European countries [17,18]. However, there is no evidence for the existing inequities in dental caries in Roma children, although this population is the most important and sizable ethnic minority in Europe comprising between 10 and 12 million [19]. Moreover, the Roma population occupies an extreme position in the social gradient with the highest levels of social exclusion relative to the general population [20]. Nonetheless, the Roma population represents approximately 2% of the total population in Spain [21]. As in the rest of Spain, the Basque Country, an autonomous region in the north of Spain, has experience an intense process of arrival of immigrant population in recent decades. This has led to new births and family reunification processes yielding an increase in the child population of immigrant origin representing 15.9% of the population younger than 24 years [22].

Despite this reality, parental migratory status inequities in childhood caries have been rarely studied. In fact, there is no study including Roma children. However, a study has examined parental migratory status inequalities in dental caries among children focusing on the effect of acculturation [23]. Furthermore, there are no studies examining inequities in dental caries in primary dentition, despite its importance in these teeth as an indicator of future caries experience [24]. Interestingly, health systems and their services can play a moderating role on the effects of the social determinants and health inequities [25]. In Spain, the dental care services offered by the public system are carried out through the Children’s Dental Care Program with different coverage and benefits across the Regions of Spain. Although this program provides free preventive and restorative care for the permanent dentition of all children aged 7 to 15 years old [26], treatment for tooth decay in the primary dentition must be paid out of pocket. The latter contributes to the existing inequities in dental caries among socioeconomically disadvantaged groups, including immigrants and ethnic minorities. Therefore, this study aims to examine the migratory status/ethnic inequities in dental caries in children aged 4 to 9 years and the impact of the Children’s Oral Health Program in the Municipality of Bilbao in the Basque Country Region.

## 2. Materials and Methods

### 2.1. Study Population and Sources of Information

A descriptive cross-sectional study was conducted among school children aged 4 to 9 residing in the Municipality of Bilbao, a city in north Spain. The study used data from the Children’s Oral Health Survey carried out by the Bilbao City Council (ESOBI’2017). This survey is based on the National Oral Health Survey [27] and included all children in public school in the Municipality of Bilbao. For the collection of data, 21 Public School Centers of Bilbao were selected based on their ethnic diversity using data from the Bilbao City Council as of January 2016 [28]. The ESOBI’2017 collected information via questionnaire completed by the child’s parent on sociodemographic variables, dietary habits, dental care utilization and oral health. Oral health was determined through clinical examinations conducted between June 2016 and October 2017 by a single dentist trained according to the World Health Organization guideline [29]. The examinations were conducted using a plane mouth mirror No. 5, a blunt dental probe, natural light and a halogen lamp. The ESOBI’2017 had a response rate of 89%.

### 2.2. Variables

Dental caries and their experience in primary and permanent teeth were included as dependent variables. Dental caries was determined according to the diagnostic criteria recommended by the WHO as “a lesion present in the tooth, which has a cavity, undermined enamel or a wall floor that is appreciably softened” [29]. The experience of caries was determined using the *dft* (temporary teeth) and *DMFT* (permanent teeth) indices, which represent the cumulative experience of past and current caries. These indices were calculated by adding the number of caries (d/D) and filled (f/F) for primary and permanent teeth and, additionally, the number of missing (M) in the case of permanent teeth. Finally, the presence of at least one untreated caries (caries ≥ 1) and experience of caries (*dft* index ≥ 1 and *DMFT* index ≥ 1) in primary and permanent dentitions were considered as dependent variables.

The independent variable was children’s migratory status and ethnicity categorized as Spanish (both parents born in Spain and of non-gypsy ethnicity), Spanish Roma (both parents born in Spain and of gypsy ethnicity) and immigrant (both parents born in countries with a not very high Human Development Index (HDI) or ≤0.80). Immigrants from very high HDI countries were excluded because they have a similar or better socioeconomic position than the native population, and thus, the latter may mask inequalities between native and immigrant population. Consistent with a previous study [30], in the case of mixed origins, only maternal migratory status/ethnicity was considered for the assignment to one of the three groups [15].

Consistent with previous studies [11,31,32], we considered variables associated with the exposures and/or outcomes as covariates were included in the analyses. These variables included age, continuous and categorical (4–6 and 7–9 years), and sex. The category of 4 to 6 years was considered as one recommended by the WHO for the study of the primary dentition [29] and 7 years or older as the age for access to dental care at Children’s Oral Health Program (PADI) in the Basque Country Region. For socioeconomic position, the parents’ highest level of education was included as an individual measure and neighborhood deprivation quintile was included as an aggregate measure. Education was collected by the question *What is your highest level of studies achieved?* with four response categories: no formal education and primary, secondary and university education. The responses were categorized into primary or less and secondary or higher. Socioeconomic deprivation quintile (MEDEA deprivation index) was determined in the census sections of the Basque Country Region of the school. This index combines five socioeconomic indicators in each census section (from 500 to 2000 inhabitants): proportion of unemployment in the active population, manual workers, temporary wage earners, insufficient education and insufficient education in the younger population. The index is specified in quintiles from highest to lowest deprivation [33] and further aggregated as high (Q3, Q4 and Q5) and low deprivation (Q1 and Q2). For caries-related behavioral factors, tooth brushing and diet, both self-reported, were included [31]. Tooth brushing was collected via the question *how often do you usually brush your teeth?* The responses were categorized into less than twice a day and greater or equal than twice a day. A cariogenic diet was defined from the frequency of consumption of sweets (candies) or sugary soft drinks and specified as the frequency of consumption of sweets or soft drinks of three times or more per week [34]. Finally, dental visit as an indicator of preventive oral health was defined by the question, how *often does your child go to the dentist for a check-up?* The responses were categorized as once a year or less than once a year.

Of the total study participants (*n* = 1448), we excluded those born in countries with a very high HDI (>0.80; *n* = 15), records with missing information on education (*n* = 26) and for any of the following caries-related behaviors: tooth brushing (*n* = 6), cariogenic diet (*n* = 6) and dental visits (*n* = 7). These exclusions resulted in an analytical sample of 1388, including 839 Spanish, 136 Spanish Roma and 413 immigrants.

### 2.3. Statistical Analysis

Descriptive statistics for selected characteristics and the oral health outcomes were calculated for the total population and by parental immigration status/ethnicity. Chi-squared of independence statistics were used for categorical data and the Kruskal–Wallis test for continuous data. Log binomial regression was used to quantify the association of parental immigration status/ethnicity with tooth decay for (1) the primary and the permanent dentitions, separately in children 4 to 9 years old, and (2) for permanent teeth in children aged 7 to 9 years before and after controlling for selected covariates. The analysis was repeated to estimate *dft* and *DMFT* rate ratios with Poisson regression using a robust variance estimator. Data management procedures and analyses were carried out using SPSS 24.0. (IBM, Armonk, NY, USA).

## 3. Results

Descriptive statistics for sociodemographic characteristics, caries-related behavioral factors, dental visits as well as caries and experience of caries for the study population according to migratory status/ethnicity are presented in Table 1. More than half of the sample were children ages 4 to 6 years and boys. Compared with children of Spanish parents, children with Spanish Roma and immigrant parents were less likely to have secondary or higher studies. Roma children were more likely to be in a high school district deprivation. Regarding oral-caries-related health behaviors, Spanish Roma and immigrant children reported a higher proportion of having a cariogenic diet compared with Spanish children, whereas Spanish Roma reported a higher proportion of brushing their teeth less than two times per day. Compared with Spanish Children, Spanish Roma and immigrant children were less likely to have had a dental visit in the past year.

The prevalence of dental caries in the study population was 35.4%, with the higher burden being carry by the Roma population with 20.6%. Overall, caries status was worse in Spanish Roma and immigrant’s children compared with Spanish Children. In primary teeth, the proportion of Spanish children with at least one caries was 24.1%, while for Spanish Roma and Immigrants children, these proportions were 70.6% and 42.6%, respectively (*p* < 0.001). For untreated tooth decay experience (*dft* ≥ 1), the proportions were higher in Spanish and immigrant children’s (27.8% and 45%, respectively), but the highest proportion was observed in Spanish Roma children (70.6%; *p* < 0.001). In permanent teeth, the proportion of Spanish children with at least one caries was 2.4%, while in Spanish Roma children and immigrant children, these proportions were 24.3% and 5.6%, respectively (*p* < 0.001). For permanent teeth, the proportion of caries experience (*DMFT* ≥ 1) in Spanish children was 3.3%, while in Spanish Roma children and immigrant children, these proportions were 25.0% and 6.1%, respectively (*p* < 0.001).

Table 2 shows the unadjusted and adjusted prevalence ratios (PRs) and 95% confidence intervals (CI) for dental caries outcomes in children for parental immigration status/ethnicity. In primary teeth, Spanish Roma and immigrant children had 2.93 (95% CI: 2.49–3.45) and 1.77 (95% CI: 1.50–2.09) higher probabilities of having at least 1 tooth affected with dental caries compared with Spanish Children. In Spanish Roma children, the probabilities were reduced (PR: 2.30, 95% CI: 1.87–2.82) after adjusting for parents’ educational attainment, school district socioeconomic deprivation, brushing teeth less than two times per day, having a cariogenic diet at least three times per week and a dental visit in the past year. However, among immigrant children, the probability of having at least one tooth with dental caries remained nearly identical after adjustment (PR: 1.74, 95% CI: 1.47–2.07). Similarly, for caries experience in the primary dentition (*dft* ≥ 1), the probability was higher in Spanish Roma and immigrant children (PR: 2.54, 95% CI: 2.18–2.96; and PR: 1.62, 95% CI: 1.39–1.89, respectively) compared with Spanish children. After adjusting for selected variables, the associations were attenuated in Spanish Roma (PR: 2.04, 95% CI: 1.68–2.48) but barely changed in immigrant children (PR: 1.63, 95% CI: 1.39–1.91). In permanent teeth, Spanish Roma and immigrant children had 10.18 (95% CI: 6.02–17.21) and 2.34 (95% CI 1.30, 4.20) higher probability of having at least 1 tooth with dental caries compared with Spanish Children. After adjusting for selected variables, these probabilities were reduced in both, Spanish Roma (PR: 6.12; 95% CI: 3.19–11.74) and in immigrant children (PR: 2.13; 95% CI: 1.14–3.97). Finally, for untreated tooth decay experience (*DMFT* index ≥ 1), the probabilities were significantly higher in Spanish Roma and immigrant children (PR: 7.48; 95% CI: 4.70–11.94 and PR: 1.81; 95% CI: 1.07–3.07, respectively) compared with Spanish children. After adjustment, the probabilities were reduced in both, Spanish Roma (PR: 4.83; 95% CI: 2.60–8.98) and immigrant children (PR: 1.74; 95% CI: 1.00–3.03).

Appendix A Table A1 presents the unadjusted and adjusted rate ratios (RRs) and 95% CIs for untreated tooth decay experience (*dft* and *DMFT* index ≥ 1) in children for parental immigrant origin/ethnicity. Compared with Spanish children, Spanish Roma and immigrant children had a higher rate of caries experience in primary and permanent dentition. After adjusting for parents’ educational attainment, school district socioeconomic deprivation, brushing teeth less than two times per day, a cariogenic diet and dental visits in the past year, the probabilities were reduced in the Spanish Roma and immigrant children. The results of the RRs were consistent with the PRs.

Table 3 shows the unadjusted and adjusted prevalence ratios (PRs) and 95% CIs for dental caries in permanent teeth in children aged 7 to 9 years for parental migratory/ethnic status. Spanish Roma children had 8.40 (95% CI: 5.09–13.86) higher probability of having at least one caries compared with Spanish Children. After adjusting for parents’ educational attainment, school district socioeconomic deprivation, brushing teeth less than one times per day, having cariogenic diet and a dental visit in the past year, the differences were attenuated (PR: 6.20; 95% CI: 3.18–12.12) but remained significant. For untreated tooth decay experience in the permanent dentition (*DMFT* index ≥ 1), the probability remained higher in Spanish Roma children (PR: 6.00; 95% CI: 3.86–9.34), but the association was attenuated after adjusting for all variables (PR: 4.52; 95% CI: 2.46–8.32). However, these associations were not significant for immigrant children.

## 4. Discussion

The findings of this study show a higher probability of dental caries and untreated tooth decay in both primary and permanent dentitions, among Spanish Roma and immigrant children compared with Spanish children, after adjusting for demographic and socioeconomic characteristics, caries-related behaviors and dental visits. Specifically, for ages of 7 to 9 years, there were no differences in the probability of dental caries and the experience of untreated tooth decay in the permanent dentition in immigrant children relative to Spanish children. However, in the Spanish Roma, although the odds were reduced, they remained significantly higher.

The higher prevalence of dental caries and untreated tooth decay in primary and permanent dentitions in immigrant children compared with Spanish children is consistent with previous studies both in Spain [23] and in Europe [9,11,15,31]. Studies conducted in Slovakia and Serbia [35,36] also show higher prevalence of tooth decay in Spanish Roma children. In addition, in the case of Spanish Roma children, it is important to point out that in primary teeth, the proportions of dental caries and untreated tooth decay experience were equal, which confirms the low use of preventive and restorative dental visits in this population [35]. This finding suggests that the consequences of belonging to this ethnic minority begin early in life. On the other hand, the inequities in dental caries observed in both, primary and permanent dentitions, are consistent with studies conducted in the Netherlands [15], Sweden [9,16] and Canada [31], in which inequities also persist, regardless of socioeconomic status, caries-related behaviors and dental visits. Hence, from the first years of life, immigrant status and ethnic background may be considered as a determinant of inequities for oral health outcomes.

Despite the persistence of caries inequities in children with an immigrant background in countries with universal dental health programs [9], we found that the inequities in dental caries outcome disappeared in immigrant children aged 7 to 9 years compared with Spanish children. This finding can be explained by the role of the Children’s Dental Care Program in the Basque Country that begins at age 7 and ends at age 15. This program has different characteristics compared with other dental health programs in other regions of Spain and Europe. Specifically, this program has an equity approach, with a stable financial endowment over the last 30 years, a wide healthcare network with free choice of dentist, not restricted to public providers and the annual active recruitment of children [37]. These characteristics have made it possible to reduce or eliminate both economic and/or geographical access barriers, facilitating the use of dental services to the most disadvantaged and needed groups as well as those with greater access barriers, such as immigrant children. In fact, it is estimated that only between 7% and 9% of children with the benefits of the program did not use the services [38,39]. This finding confirms the role that health services with an equity approach can play in serving as a safety net for the social determinants that generate health inequities [25].

In Roma children, unlike in immigrant children, tooth decay inequities at the age of 7 to 9 years persisted, indicating that they have not benefited from the Children’s Dental Care Program like the rest of the children in the Basque Country. Although there are no studies on inequities in dental caries in Roma children, our findings are consistent with studies conducted with Roma community in Spain and Europe showing worse health indicators [40] and a lower use of health resources even in countries with universal health systems [41]. The high levels of social exclusion in employment, housing or education and the stigma and structural and interpersonal discrimination suffer by the Roma population could also explain the low utilization of the Children’s Dental Care Program.

Our study presents several limitations that must considered. First, it is a cross-sectional study that does not allow a temporal relationship between exposure and outcome to be established. However, immigrant status and ethnicity precede the outcomes of interest. Second, our study used a clinical examination without x-rays. The latter could have led to an underestimation of dental caries in our findings, especially for proximal surfaces for posterior teeth. Third, the non-disaggregation by country of birth of the parents, which did not allow the examination of differences in dental caries outcomes of immigrant children according to the country of origin of the parents. The lack of this information may skew the results by over or underestimating the inequities in our study between immigrant and Spanish children. Finally, despite the small sample for Spanish Roma, our sample represents the proportional of these groups in the school child population of the municipality of Bilbao [42]: <10% Spanish Roma. Despite the aforementioned limitations and to the best of our knowledge, this study is the first in Spain to examine inequities in cavities and untreated tooth decay experience in children aged 4 to 9 according to the migratory status of their parents and the only one that includes children of the Roma ethnic group. In addition, dental caries was determined by a dental examination in which all Public School Centers with ethnic diversity and/or immigrant origin of more than 30% of the Municipality of Bilbao participated.

## 5. Conclusions

Our findings contribute to our understanding of inequities in dental caries and untreated tooth decay in immigrant and ethnic minority children, an issue rarely studied in our context. The findings show that parental immigration status and ethnicity affect dental caries outcomes in immigrant and Roma children in both primary and permanent dentitions. In addition, our study calls attention to the need to expand the Children’s Dental Care Program for the primary dentition. Finally, and in the case of Roma children, it is necessary to apply new inclusive strategies in dental health services centered not only on the socioeconomic situation of this population but also on their beliefs and behaviors related to health and healthcare utilization. The latter could contribute to prevent and/or minimize the negative consequences of dental caries for their present oral health and beyond. In this way, both the disease burden and dental health inequities in Roma children could be reduced and/or eliminated.

## Figures and Tables

**Table 1:** Distribution of selected characteristics for participants according to parental migratory status/ethnicity: Children’s Oral Health Survey, Municipality of Bilbao, 2017.

	Spanish *n* = 839	Spanish Roma *n* = 136	Immigrant *n* = 413	Total *n* = 1388	*p*-Value *
	%(*n*)	%(*n*)	%(*n*)	%(*n*)	
**Age (years)**					0.359
4–6	56.6 (475)	52.2 (71)	59.1 (244)	56.9 (790)	
7–9	43.4 (364)	47.8 (65)	41.0 (170)	43.1 (598)	
**Sex**					0.150
Female	45.5 (382)	46.3 (63)	51.3 (212)	47.3 (657)	
Male	54.5 (457)	53.7 (73)	48.7 (201)	52.7 (731)	
**Parents’ educational attainment**					<0.001
Primary or less	18.1 (152)	91.2 (124)	33.9 (140)	30.0 (416)	
Secondary or higher	81.9 (687)	8.8 (12)	66.1 (273)	70.0 (972)	
**School district deprivation**					<0.001
High (Q3–Q5)	67.5 (566)	86.0 (117)	58.6 (242)	66.6 (925)	
Low (Q1–Q2)	32.5 (273)	14.0 (19)	41.4 (171)	33.4 (463)	
**Caries-related behaviors**					
Brushing teeth <2 times per day	20.1 (169)	38.2 (52)	17.7 (73)	21.2 (294)	<0.001
Cariogenic diet ≥3 times per week	49.3 (414)	77.2 (105)	55.4 (229)	53.9 (748)	<0.001
**At least a dental visit in the past year**	34.1 (286)	53.7 (73)	53.7 (222)	41.9 (581)	<0.001
**Caries in primary teeth**					
Mean (SD)	0.6 (1.40)	2.5 (2.4)	1.5 (2.5)	1.0 (2.01)	<0.001
Caries ≥ 1 (%)	24.1(202)	70.6 (96)	42.6 (176)	34.1 (474)	<0.001
***dft* index ^a^**					
Mean (SD)	0.7 (1.5)	2.5 (2.4)	1.7 (2.6)	1.2 (2.08)	<0.001
Index ≥ 1	27.8 (233)	70.6 (96)	45.0 (186)	37.1 (515)	<0.001
**Caries in permanent teeth**					
Mean (SD)	0.0 (0.2)	0.5 (1.8)	0.1 (0.4)	0.1 (0.5)	<0.001
Caries ≥ 1 (%)	2.4 (20)	24.3 (33)	5.6 (23)	5.5 (76)	<0.001
***DMFT* index ^b^**					
Mean (SD)	0.0 (0.28)	0.6 (1.20)	0.1 (0,42)	0.1 (0.51)	<0.001
Index ≥ 1	3.3 (28)	25.0 (34)	6.1 (25)	6.3 (87)	<0.001

^a^*dft* index: decayed and filled teeth for the primary teeth; ^b^ *DMFT* index: decayed, missing and filled teeth for the permanent teeth; * *p*-values are from Chi-squared statistics for categorical variables as well as ANOVA and Kruskal–Wallis statistics for continuous variables.

**Table 2:** Prevalence Ratios and their 95% Confidence Intervals of dental caries outcomes for parental migratory status/ethnicity: Children’s Oral Health Survey, Municipality of Bilbao, 2017.

	Unadjusted	Model 1	Model 2
**Caries in primary teeth ≥ 1**			
Spanish	1.00	1.00	1.00
Spanish Roma	2.93 (2.49–3.45)	2.88 (2.45–3.39)	2.30 (1.87–2.82)
Immigrant	1.77 (1.50–2.09)	1.78 (1.51–2.09)	1.74 (1.47–2.07)
***dft* ≥ 1**			
Spanish	1.00	1.00	1.00
Spanish Roma	2.54 (2.18–2.96)	2.49 (2.13–2.91)	2.04 (1.68–2.48)
Immigrant	1.62 (1.39–1.89)	1.63 (1.40–1.90)	1.63 (1.39–1.91)
**Caries in permanent teeth ≥ 1**			
Spanish	1.00	1.00	1.00
Spanish Roma	10.18 (6.02–17.21)	7.44 (4.37–12.70)	6.12 (3.19–11.74)
Immigrant	2.34 (1.30–4.20)	2.29 (1.27–4.12)	2.13 (1.14–3.97)
***DMFT* ≥ 1**			
Spanish	1.00	1.00	1.00
Spanish Roma	7.49 (4.70–11.94)	5.37 (3.33–8.68)	4.83 (2.60–8.98)
Immigrant	1.81 (1.07–3.07)	1.76 (1.04–2.98)	1.74 (1.00–3.03)

*dft* index: decayed and filled teeth for the primary teeth; *DMFT* index: decayed, missing and filled teeth for the permanent teeth; Model 1: Adjusted for sex and age (continuous); Model 2: additionally, adjusted for parents’ educational attainment, School District Deprivation, brushing teeth <2 times per day, having cariogenic diet ≥3 times per week and at least 1 dental visit in the past year.

**Table 3:** Prevalence Ratios and their 95% Confidence Intervals of dental caries outcomes in permanent teeth in children aged 7 to 9 years for parental migratory status/ethnicity: Children’s Oral Health Survey, Municipality of Bilbao, 2017.

	Unadjusted	Model 1	Model 2
**Caries in permanent teeth ≥1**			
Spanish	1.00	1.00	1.00
Spanish Roma	8.40 (5.09–13.86)	8.27 (4.96–13.79)	6.20 (3.18–12.12)
Immigrant	1.61 (0.85–3.08)	1.60 (0.83–3.04)	1.28 (0.63–2.60)
***DMFT* ≥ 1**			
Spanish	1.00	1.00	1.00
Spanish Roma	6.00 (3.86–9.34)	5.81 (3.64–9.26)	4.52 (2.46–8.32)
Immigrant	1.31 (0.74–2.32)	1.28 (0.72–2.28)	1.11 (0.59–2.08)

*DMFT* index: decayed, missing, and filled teeth for the permanent teeth; Model 1: Adjusted for sex and age (continuous); Model 2: additionally, adjusted for parents’ educational attainment, School District Deprivation, brushing teeth <2 times per day, having cariogenic diet ≥3 times per week and at least 1 dental visit in the past year.

## Appendix A

**Table A1:** Rate Ratios and their 95% Confidence Intervals of dental caries outcomes for parental migratory status/ethnicity: Children’s Oral Health Survey, Municipality of Bilbao, 2017.

	Unadjusted	Model 1	Model 2
		***dft***	
Spanish	1.00	1.00	1.00
Spanish Roma	3.51 (2.81–4.38)	3.44 (2.76–4.30)	2.66 (2.03–3.48)
Immigrant	2.38 (1.93–2.93)	2.41 (1.95–2.96)	2.36 (1.90–2.94)
		***DMFT***	
Spanish	1.00	1.00	1.00
Spanish Roma	12.34 (7.19–21.17)	8.49 (4.99–14.44)	7.13 (3.94–12.91)
Immigrant	1.98 (1.08–3.63)	1.90 (1.04–3.48)	1.83 (1.02–3.29)

*dft* index: decayed and filled teeth for the primary teeth; *DMFT* index: decayed, missing and filled teeth for the permanent teeth; Model 1: Adjusted for sex and age (continuous); Model 2: additionally, adjusted for parents’ educational attainment, School District Deprivation, brushing teeth <2 times per day, having a cariogenic diet ≥3 times per week and at least 1 dental visit in the past year.
